# The impact of physical activity on adolescent social anxiety: a growth curve model analysis

**DOI:** 10.3389/fpsyg.2026.1862581

**Published:** 2026-07-31

**Authors:** Rui Qin, Hongli Wu, Jihe Zhou, Xia Huang, Shuai Wang

**Affiliations:** School of Sports Medicine and Health, Chengdu Institute of Physical Education, Chengdu, Sichuan, China

**Keywords:** adolescents, latent growth, nonlinear, physical activity, social anxiety

## Abstract

**Objective:**

This study examined the dynamic association between adolescents’ physical activity levels and social anxiety using a latent growth curve model.

**Methods:**

A longitudinal survey was conducted with 851 students from five middle schools in Sichuan, Jiangsu, and Guangdong provinces, China. Assessments were conducted at 3-month intervals. Physical activity and social anxiety were measured using the IPAQ and the Social Anxiety Scale for Adolescents. Latent growth curve models were estimated using Mplus 8.0.

**Results:**

(1) Physical activity followed a nonlinear trend, initially decreasing and then increasing (initial level = 3.267, *p* < 0.01; linear rate = −0.087, *p* < 0.01; nonlinear rate = 0.026, *p* < 0.01). (2) Social anxiety showed an “increase-then-decrease” trajectory (initial level = 2.713, *p* < 0.01; linear rate = 0.178, *p* < 0.01; nonlinear rate = −0.064, *p* < 0.01). (3) The initial level of physical activity negatively predicted social anxiety’s initial level (β = −0.250, *p* < 0.001) and change rate (β = −0.154, *p* < 0.001).

**Conclusion:**

Higher physical activity levels were associated with lower social anxiety and slower increases in social anxiety over time. Promoting regular physical activity may help support adolescent mental health.

## 1 Introduction

Evolutionary psychology suggests that forming strong social connections is a fundamental human need for adapting to the environment and is closely related to individual survival and development (Gautam et al., 2024; Yang et al., 2024). From this perspective, sensitivity to social evaluation may function as an adaptive mechanism that helps individuals monitor acceptance or rejection within peer groups. However, excessive fear of negative evaluation in social contexts may increase the risk of social anxiety among adolescents (Fredrick and Luebbe, 2024; Villalongo Andino et al., 2024). Social anxiety is a common psychological problem characterized by excessive worry and avoidance in social situations. It may adversely affect adolescents’ academic performance and peer relationships and may increase the risk of long-term mental health problems (Flick et al., 2024; Kajastus et al., 2024; Mou et al., 2024; Urbán et al., 2024). Thus, researching social anxiety is both theoretically and practically significant. From an evolutionary psychology perspective, regular movement and social cooperation are closely related to humans’ evolved adaptive needs, whereas modern sedentary lifestyles may create a mismatch between adolescents’ biological needs for movement and their daily behavioral patterns. Based on this perspective, together with social support theory, self-efficacy theory, and Self-Determination Theory, this study assumes a clearer conceptual pathway: physical exercise may first promote neurobiological regulation, such as reducing physiological arousal and regulating neurotransmitter activity; these changes may then facilitate psychological adjustment, including improved self-efficacy and emotion regulation; finally, these psychological changes, together with increased peer interaction and perceived social support, may contribute to reduced social anxiety.

In recent years, increasing attention has been paid to the potential role of physical activity in reducing social anxiety. Previous studies have suggested that physical activity may reduce anxiety by improving emotional regulation, self-efficacy, and social support (Cécillon et al., 2024; Fang and Mushtaque, 2024; Hayatipoor et al., 2024). There is a growing body of research focusing on the positive effects of physical activity in alleviating social anxiety among adolescents. physical activity has been associated with lower overall anxiety and with reduced social anxiety symptoms (Chen S. et al., 2024; Ren and Li, 2020). Evidence from another cultural context similarly suggests a negative association between physical activity and social anxiety (Solmaz et al., 2025). Following the pathway proposed above, physical activity may first induce neurobiological changes by modulating neurochemical systems, including neurotransmitters (e.g., dopamine and serotonin) and endogenous opioids (e.g., endorphins), thereby reducing physiological arousal and improving anxiety states (Gaudlitz et al., 2015; Hossain et al., 2024; Mu et al., 2024; Stone, 2018). These neurobiological changes may further support psychological changes, including improved self-efficacy and emotion regulation, which help adolescents cope with perceived negative evaluation in social situations (Singh et al., 2023; Zhang et al., 2022). In addition, physical activity, particularly team sports, may provide opportunities for social interaction, strengthen peer connections, and enhance perceived social support, which may further reduce social anxiety (Jetten et al., 2022). Within the evolutionary psychology framework, these activities may help satisfy adolescents’ evolved needs for movement, social belonging, and peer acceptance, thereby linking physical activity to lower social anxiety.

Despite the widely acknowledged benefits of physical activity in reducing adolescent social anxiety, existing research has limitations in design and methodology. Many studies employ cross-sectional designs that focus on static correlations between physical activity and social anxiety, neglecting the dynamic relationship between the two over time (Chen S. et al., 2024; Deng and Wang, 2024). Furthermore, although cross-sectional studies can describe associations between physical activity and social anxiety at a single time point, they are limited in their ability to examine temporal change, long-term developmental trajectories, and complex interactions (Ren and Li, 2020; Zhang Z. et al., 2024). Therefore, more longitudinal evidence is needed to clarify how general physical activity participation changes over time and how its developmental trajectory is dynamically associated with changes in adolescent social anxiety.

Although the above theories provide the conceptual basis for understanding how physical activity may influence adolescent social anxiety, latent growth curve modeling (LGCM) serves as a methodological tool for examining their dynamic associations over time. LGCM allows for the systematic examination of the initial levels, change trends, and interrelationships of physical activity and social anxiety through multi-time point tracking data (Li X. et al., 2024). Studies utilizing linear and nonlinear unconditional latent growth models for physical activity have found that participation levels typically exhibit a stable linear growth trend (Kim et al., 2021), which is closely associated with improvements in mental health (Bae et al., 2024). On the other hand, the trajectory of social anxiety may show nonlinear characteristics, such as a steep initial decline followed by a more gradual decrease, a pattern that may be closely related to individual emotional regulation capabilities and external environmental support (Li X. et al., 2024).

Multivariate Latent Growth Curve Modeling (Multi-LGCM) can model the developmental trajectories of physical activity and social anxiety simultaneously, examining their dynamic association over time through the analysis of correlation coefficient matrices, key parameter estimation, and fit indices. For instance, research has found that the initial level of physical activity can significantly predict the initial level of social anxiety, and the rate of change in physical activity can significantly predict the downward trend in social anxiety, indicating a close negative correlation between the two in the dynamic change process (Ren and Li, 2020). Moreover, the multivariate developmental trajectories of physical activity and social anxiety may exhibit synchronicity, such as a rapid decrease in social anxiety levels coinciding with a rapid increase in physical activity participation levels (Meyerhoff et al., 2021).

Building on this theoretical framework and empirical literature, the present study used a multi-time-point longitudinal design and applied latent growth curve modeling (LGCM) and multivariate latent growth curve modeling (MLGCM) to examine the developmental trajectories of adolescent physical activity and social anxiety and their dynamic associations. Within this framework, physical activity was regarded as a potential protective factor that may reduce social anxiety through a progressive pathway from neurobiological regulation to psychological adjustment and then to social anxiety reduction. The study focused on the correlation coefficient matrices, key parameter estimation of linear and nonlinear unconditional growth models, and fit indices for both, to explore the temporal dynamic characteristics and possible explanatory pathways of the alleviating effect of physical activity on adolescent social anxiety. The findings may provide theoretical and practical guidance for using physical activity as a potential intervention to reduce adolescent social anxiety.

Based on the above framework, we hypothesized that: (H1) adolescents’ physical activity would be negatively associated with social anxiety over time; and (H2) higher initial levels and more favorable growth (i.e., less decline/increase over time) in physical activity would predict lower initial levels and a slower increase in social anxiety in the parallel-process growth model.

## 2 Participants and methods

### 2.1 Participants

This study recruited Grade 7 and Grade 8 students (i.e., the first and second year of junior secondary/middle school) from five schools in Leshan, Sichuan Province; Yancheng, Jiangsu Province; and Guangzhou, Guangdong Province, China. These locations were purposively selected to capture regional diversity in China (western, eastern, and southern regions) and to include schools from areas with differing social and educational contexts. The aim was to improve the heterogeneity and generalizability of the sample rather than to conduct formal between-region comparisons. The study protocol was approved by the Ethics Review Board of Chengdu Sports University (Approval No. CDSU2025-139).

Starting in September 2023, the initial survey distributed 1,426 questionnaires and obtained 1,043 valid responses, including 652 males (62.51%) and 391 females (37.49%). The remaining questionnaires (*n* = 383) were considered invalid primarily due to substantial missing data and/or obvious response-quality issues (e.g., inconsistent or patterned responses), and therefore did not meet the pre-specified data quality criteria for analysis. At baseline (T1), the number of participants recruited from each school was: School 1 (*n* = 206), School 2 (*n* = 208), School 3 (*n* = 226), School 4 (*n* = 192), and School 5 (*n* = 211). Eligible participants were middle school students without reported major physical or mental health conditions, as determined by self-report in the questionnaire and confirmation by the school/class teachers based on routine school information; no medical records were accessed for this study. Informed consent was obtained from the schools and parents or guardians. Retention rates were 92.71% for the second survey (967 valid responses), 92.66% for the third (896 valid responses), and 91.95% for the fourth (851 valid responses). These follow-up surveys were conducted in the same cohort of students recruited at T1, and no new participants were added at T2–T4. The average age of the 851 participants, based on the fourth survey, was 14.62 years (*SD* = 0.48).

To assess the impact of sample attrition on data structure, independent sample t-tests and Levene’s tests were conducted on gender ratios, key variable means, and questionnaire scores across all data points, showing no significant differences (*P* > 0.05). Comparisons between completers and dropouts also revealed no significant differences in key variables (*P* > 0.05), indicating that sample attrition did not substantially affect the overall data structure. Data quality was closely monitored by research team members during each survey to ensure questionnaire integrity. Participants voluntarily took part in the study and received mental health education materials as feedback after each survey.

### 2.2 Research instruments

#### 2.2.1 Physical activity questionnaire

The International Physical Activity Questionnaire (IPAQ) adolescent version was used to assess participants’ physical activity. In this study, the total IPAQ score was used as an indicator of general physical activity level rather than as separate indicators of activity type, intensity, or frequency. The IPAQ has been validated in several countries and is widely used to assess physical activity across different populations (Vasheghani-Farahani et al., 2011). The Chinese version of the IPAQ for adolescents includes seven items regarding the frequency and duration of moderate and vigorous physical activities as well as walking, covering the frequency and duration of light, moderate, and vigorous activities. The formula for calculating the total MET score is: Total MET score = (High-intensity activity days × High-intensity activity minutes per day × 8.0) + (Moderate-intensity activity days × Moderate-intensity activity minutes per day × 4.0) + (Walking days × Walking minutes per day × 3.3). The IPAQ score ranges from 0 to 28, with higher scores indicating higher levels of physical activity. To ensure the applicability and reliability of the measurement, the questionnaire was pretested on a small scale before formal administration. Confirmatory factor analysis of a single-factor model indicated good structural validity (χ^2^/df = 3.78, GFI = 0.91, AGFI = 0.90, RMSEA = 0.058). Internal consistency reliability analysis across the four time points showed Cronbach’s α coefficients of 0.88, 0.86, 0.84, and 0.82 for the IPAQ, demonstrating good internal consistency and stability of the questionnaire. Nevertheless, the IPAQ is a self-report instrument and may be subject to recall and social desirability bias, particularly in adolescents; objective measures (e.g., accelerometry) should be considered in future research to improve measurement precision.

#### 2.2.2 Social anxiety scale

The Social Anxiety Scale for Adolescents (SAS-A) was used to measure the social anxiety levels of the participants. Initially developed by Finnish scholars, the SAS-A has undergone measurement invariance validation in countries such as the United States, Spain, and China, showing good psychometric properties (Ranta et al., 2012). The scale consists of 18 items divided into three dimensions: Fear of Negative Evaluation (FNE), Social Avoidance and Distress (SAD), and Anxiety in New Social Situations (ANS). The scale uses a 5-point Likert scoring system, ranging from “1 = Not at all characteristic of me” to “5 = Extremely characteristic of me,” with a total score range of 18 to 90 points, and higher scores indicating higher levels of social anxiety. To enhance the cultural adaptability of the scale, some items were localized to better suit the Chinese adolescent population. This localization was reviewed by subject-matter experts and pilot-tested to ensure clarity and age-appropriateness before formal data collection. In addition, longitudinal measurement invariance was evaluated across the four waves to support the comparability of SAS-A scores over time. Confirmatory factor analysis of a three-factor model showed good structural validity (χ^2^/df = 3.65, GFI = 0.93, AGFI = 0.92, RMSEA = 0.057). Internal consistency analysis at the four time points revealed Cronbach’s α coefficients of 0.89, 0.91, 0.88, and 0.86 for the SAS-A, indicating good internal consistency and longitudinal stability.

### 2.3 Data analysis

Data analysis was conducted in several steps. First, SPSS 26.0 was used to perform descriptive statistical analyses, including the calculation of mean, standard deviation, skewness, and kurtosis (Ranta et al., 2012). Univariate normality was assessed using skewness and kurtosis. Multivariate normality was evaluated approximately by inspecting the distribution of model residuals and checking for extreme outliers. Given the large sample size, minor deviations from normality were not expected to materially affect inference under maximum likelihood estimation. Subsequently, Mplus 8.0 software was employed to construct latent growth curve models (LGCM) to explore the developmental trajectories of adolescent physical activity and social anxiety. Confirmatory factor analysis (CFA) was conducted to validate the measurement models of physical activity and social anxiety (Gunzler and Morris, 2015). Model fit was evaluated using fit indices such as CFI, TLI, RMSEA, and SRMR, and measurement invariance was tested (Marcoulides and Yuan, 2017) to ensure the comparability of the latent variables across different time points. Additionally, linear and nonlinear unconditional latent growth models (Sterba, 2014) were constructed for both physical activity and social anxiety to analyze their initial levels (Intercept), change rates (Slope), and inter-individual differences (Variance) (Duncan et al., 2013). For nonlinear change trends, a quadratic term (Yin et al., 2024) was further added to the model to capture the nonlinear developmental characteristics of the latent variables, and the model’s fit was judged by fit indices.

Finally, based on the univariate growth models, a multivariate latent growth curve model (MLGCM) (Duncan et al., 1998) was constructed to further explore the dynamic developmental relationship between physical activity and social anxiety. In the final MLGCM, both physical activity and social anxiety were specified as quadratic growth processes (intercept, linear slope, and quadratic slope), consistent with the univariate results indicating nonlinear trajectories. Model parameters were estimated using maximum likelihood estimation (MLE) (Myung, 2003), and the statistical significance of the parameters was determined using standard error (SE), z-values, and significance levels (P-values), with overall model fit assessed using fit indices such as CFI, TLI, RMSEA, and SRMR (Marcoulides and Yuan, 2017). Missing data across waves were handled using full information maximum likelihood (FIML) under the MLE framework in Mplus, allowing all available data points to contribute to parameter estimation.

### 2.4 Research process

**FIGURE 1:**
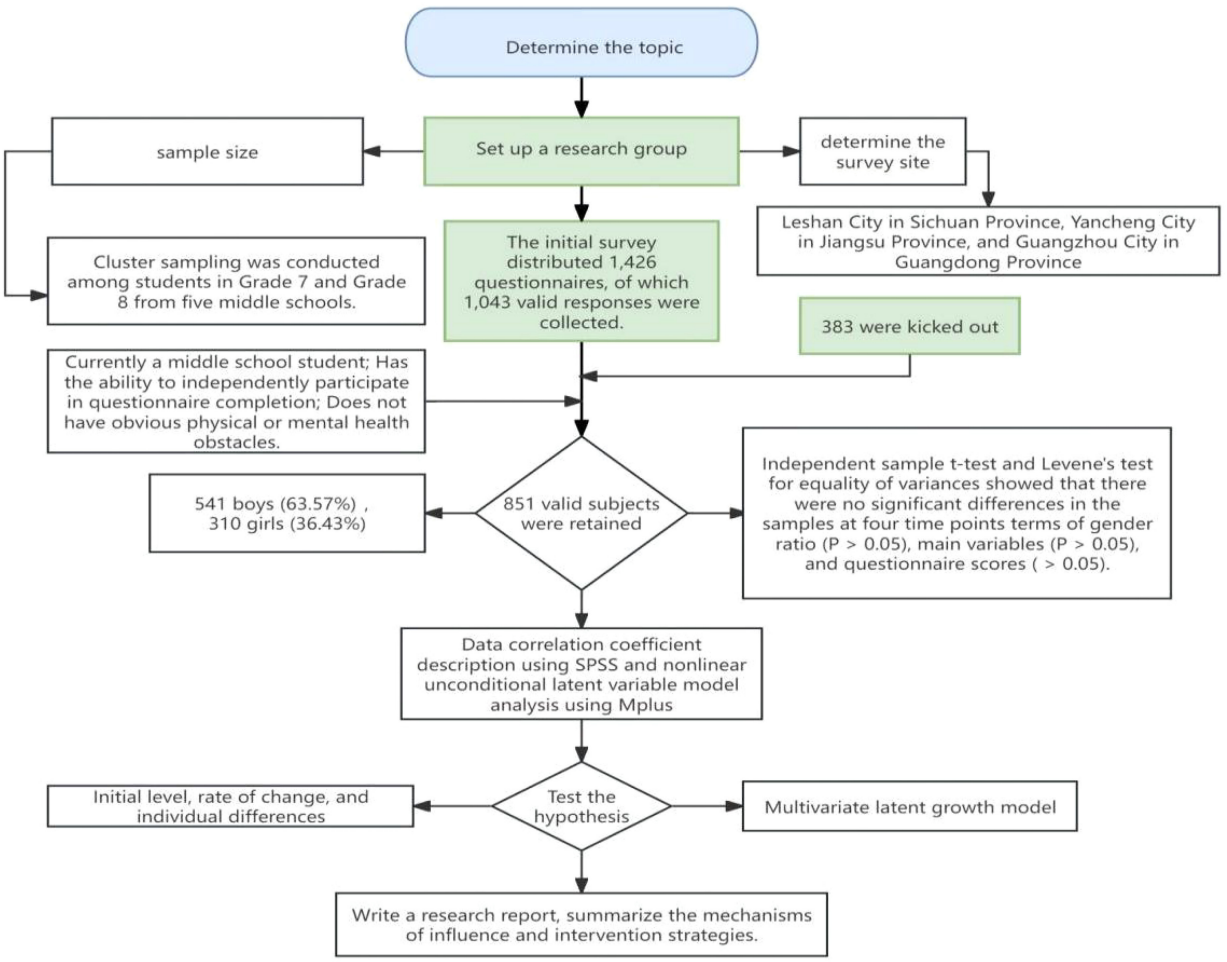
Research process.

The study employed a cluster sampling method to select students from Grade 7 and Grade 8 (i.e., the first and second year of junior secondary/middle school) in the five middle schools across the three cities, following established criteria for participant selection. After obtaining informed consent from the participants, four tracking surveys were conducted at 3-month intervals over a period of 1 year, using the International Physical Activity Questionnaire (Adolescent version) and the Social Anxiety Scale for Adolescents to measure students’ levels of physical activity and social anxiety, the order of questionnaires was randomized to reduce potential order effects. A 3-month interval was chosen to align with the school-term rhythm and to balance capturing within-year changes with minimizing recall bias and attrition; similar multi-wave longitudinal designs have been used to model temporal change in psychosocial outcomes using growth-curve/parallel-process approaches (Li X. et al., 2024; Meyerhoff et al., 2021). After data collection, SPSS and Mplus software were used for data entry, management, and analysis, constructing latent growth curve models to explore the developmental trajectories of adolescent physical activity and social anxiety and their interrelationships. Based on the analysis results, a research report was written, summarizing the mechanisms by which physical activity affects adolescent social anxiety and proposing intervention strategies. The entire research process strictly adhered to academic ethics, ensuring the protection of participant privacy. The research process is detailed in Figure 1.

## 3 Results and analysis

### 3.1 Descriptive statistics and correlation coefficient matrix

Table 1 presents the means, standard deviations, and correlation coefficient matrix for all variables. The means of physical activity across the four time points were 3.259, 3.238, 3.167, and 3.250, indicating that the participants’ levels of physical activity were generally stable, with minimal variation among individuals. The means of social anxiety across the four time points were 2.709, 2.840, 2.801, and 2.674, suggesting that the participants’ levels of social anxiety were also generally stable, but with greater individual variation compared to physical activity.

**TABLE 1:** Correlation coefficient matrix.

	*M*	*SD*	PET1	PET2	PET3	PET4	SAT1	SAT2	SAT3	SAT4
PET1	3.259	0.740	1	1	1	1	1	1	1	1
PET2	3.238	0.747	0.418**							
PET3	3.167	0.752	0.359**	0.383**						
PET4	3.250	0.775	0.362**	0.419**	0.452**					
SAT1	2.709	1.980	−0.261**	−0.131**	−0.131**	−0.148**				
SAT2	2.840	1.994	−0.176**	−0.242**	−0.141**	−0.160**	0.269**			
SAT3	2.801	1.991	−0.139**	−0.205**	−0.260**	−0.191**	0.235**	0.276**		
SAT4	2.674	1.974	−0.190**	−0.193**	−0.185**	−0.300**	0.299**	0.306**	0.358**	

Physical activity is abbreviated as PE, and Social Anxiety is abbreviated as SA. *indicates *P* < 0.05; **indicates *P* < 0.01; ***indicates *P* < 0.001.

The correlation matrix showed significant positive correlations within the same variables across the four time points (*p* < 0.01), with coefficients ranging from 0.235 to 0.452. This indicates that individuals’ levels of physical activity and social anxiety are continuous and stable across different time points. There were also significant negative correlations between different variables (*p* < 0.01), with correlation coefficients ranging from −0.131 to −0.300, indicating that higher levels of physical activity are associated with lower levels of social anxiety.

### 3.2 Trajectories of adolescent physical activity

**FIGURE 2:**
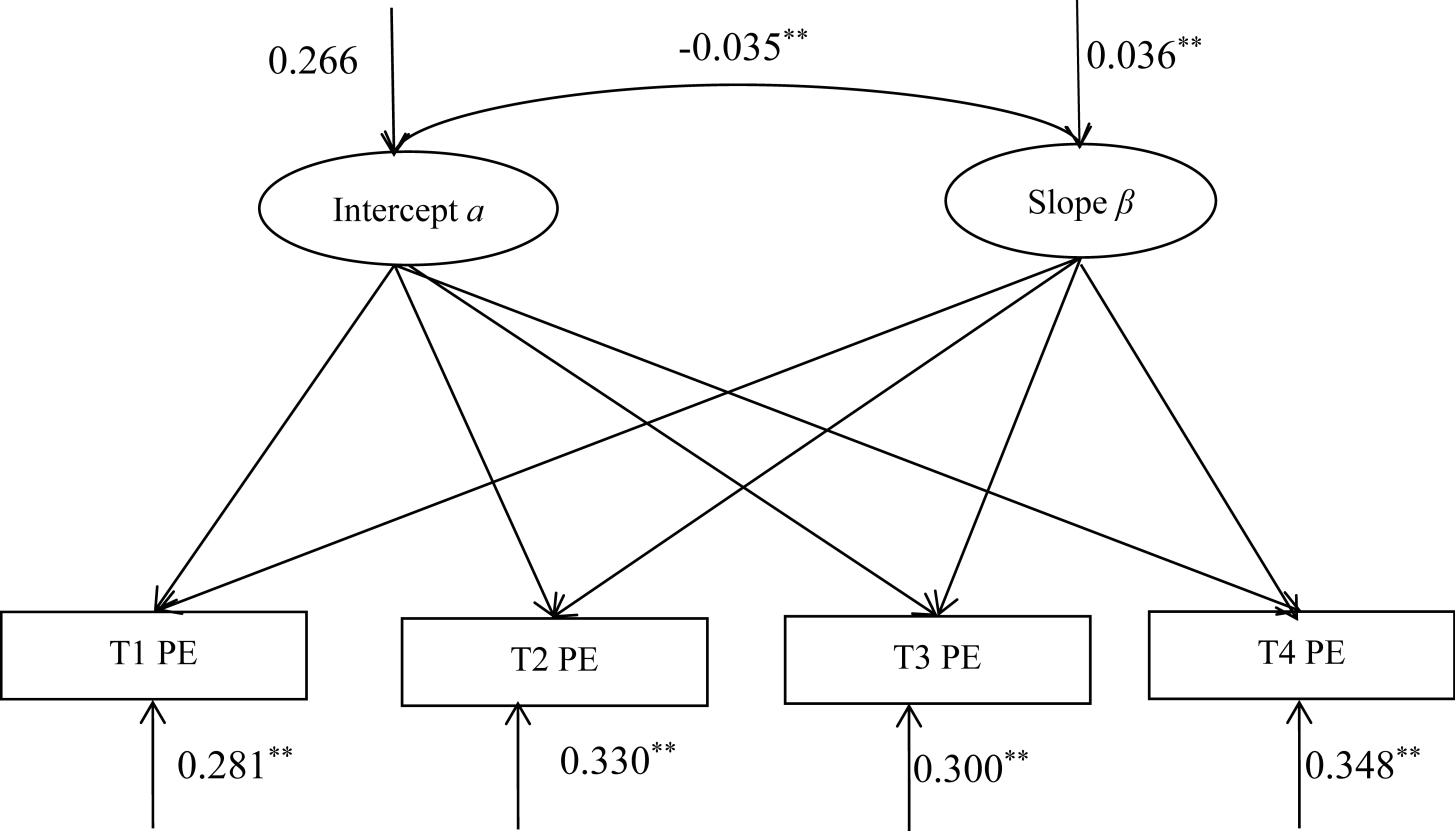
Linear developmental trajectory of adolescent physical activity. All parameters in the figure are standardized path coefficients, ** indicates *P* < 0.01, and the same applies below.

To explore the developmental trajectories of adolescent physical activity, this study employed latent growth curve modeling for analysis. As shown in Figure 2, the factor loadings of physical activity at the four time points were all above 0.51 and statistically significant (*p* < 0.01), indicating that the observed indicators contributed meaningfully to the latent construct. Overall measurement model fit was evaluated using standard fit indices (e.g., CFI, TLI, RMSEA, and SRMR), rather than factor loadings alone, and the fit indices suggested an acceptable fit.

Next, this study constructed linear and nonlinear unconditional latent growth models for physical activity to further investigate the specific features of the developmental trajectory of adolescent physical activity. As shown in Table 2, the fit indices of the linear unconditional model were good (χ^2^(10) = 14.410, *p* = 0.006, CFI = 0.989, RMSEA = 0.022, SRMR = 0.016), suggesting that the model fits the data well. The intercept of physical activity was significant (3.269, *p* < 0.01), and its variance was also significant (0.266, *p* < 0.01), indicating individual differences in initial physical activity levels. The slope was negative and significant (−0.037, *p* < 0.01), with significant variance (0.036, *p* < 0.01), suggesting a slight overall decline with individual differences in the rate of change.

**TABLE 2:** Coefficients and fit indices for linear and nonlinear unconditional latent growth models of physical activity.

Model	*x^2^ (df)*	*P*	*CFI*	*RMSEA*	*SRMR*	Coefficient			Variance		
						Intercept	Slope	Nonlinear slope	Intercept	Slope	Nonlinear slope
Linear unconditional model	14.410 (10)	0.006	0.989	0.022	0.016	3.269**	−0.037**	–	0.266**	0.036**	–
Nonlinear unconditional model	7.810 (13)	0.005	0.993	0.032	0.016	3.267**	−0.087**	0.026**	0.300**	0.061	−0.001

*indicates *P* < 0.05; **indicates *P* < 0.01; ***indicates *P* < 0.001.

**FIGURE 3:**
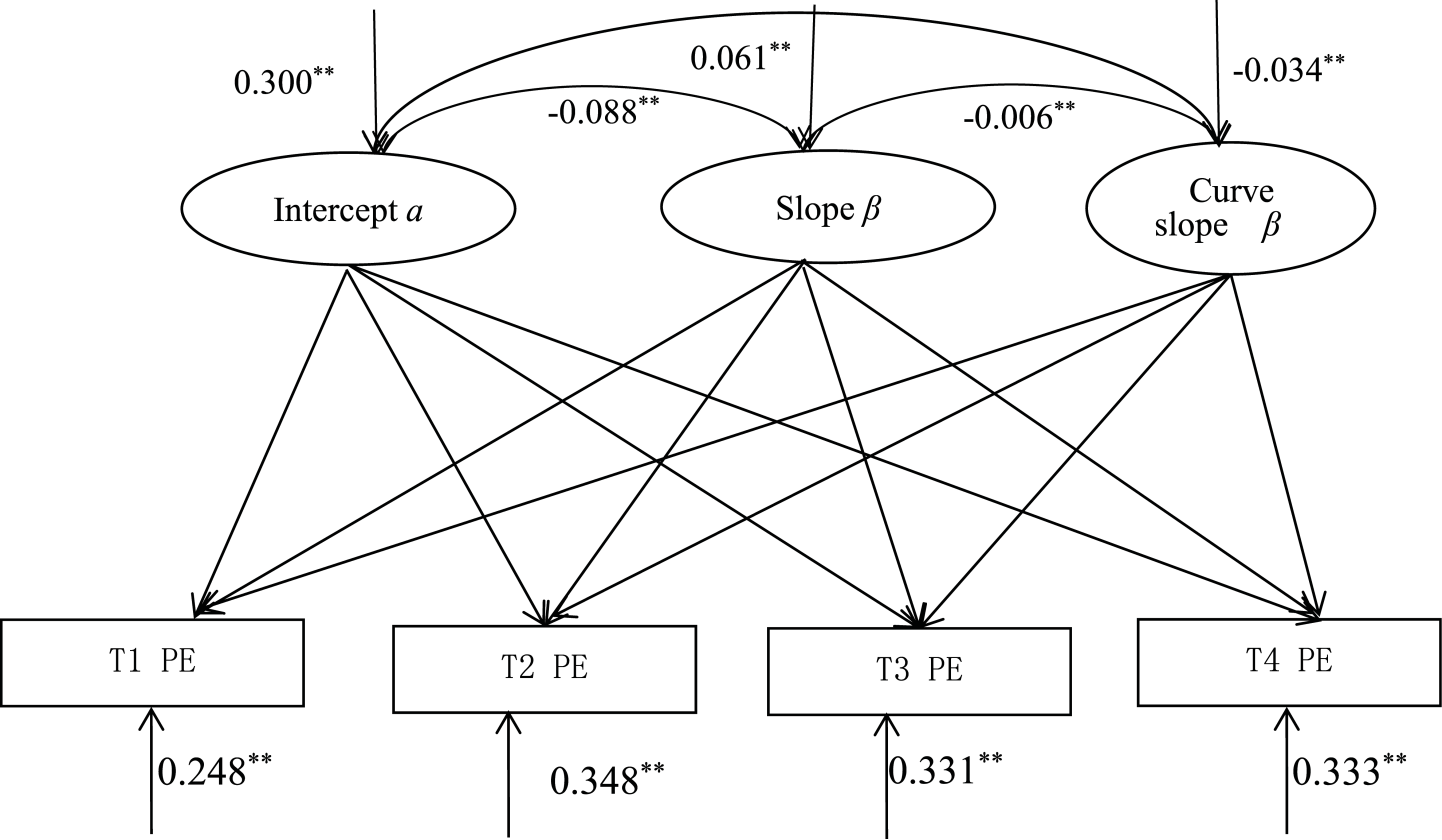
Nonlinear developmental trajectories of physical activity in adolescents. All parameters in the figure are standardized path coefficients, ** indicates *P* < 0.01, and this notation will be used throughout.

The nonlinear unconditional model also fit well (χ^2^(13) = 7.810, *p* = 0.005, CFI = 0.993, RMSEA = 0.032, SRMR = 0.016), as shown in Figure 3. The model results found that the initial level of physical activity was 3.267, which was significant and there were significant individual differences (0.300, *p* < 0.01); the linear change rate of physical activity (i.e., the slope) was −0.087, which was significant but there were no significant individual differences (0.061, *p* > 0.05); the nonlinear change rate of physical activity (i.e., the curvature slope) was 0.026, which was significant but there were also no significant individual differences (−0.001, *p* > 0.05). This indicates that the developmental trajectory of adolescent physical activity has significant nonlinear characteristics, showing a trend of decreasing and then increasing, but this nonlinear change does not significantly vary among individuals.

### 3.3 Trajectories of adolescent social anxiety

**FIGURE 4:**
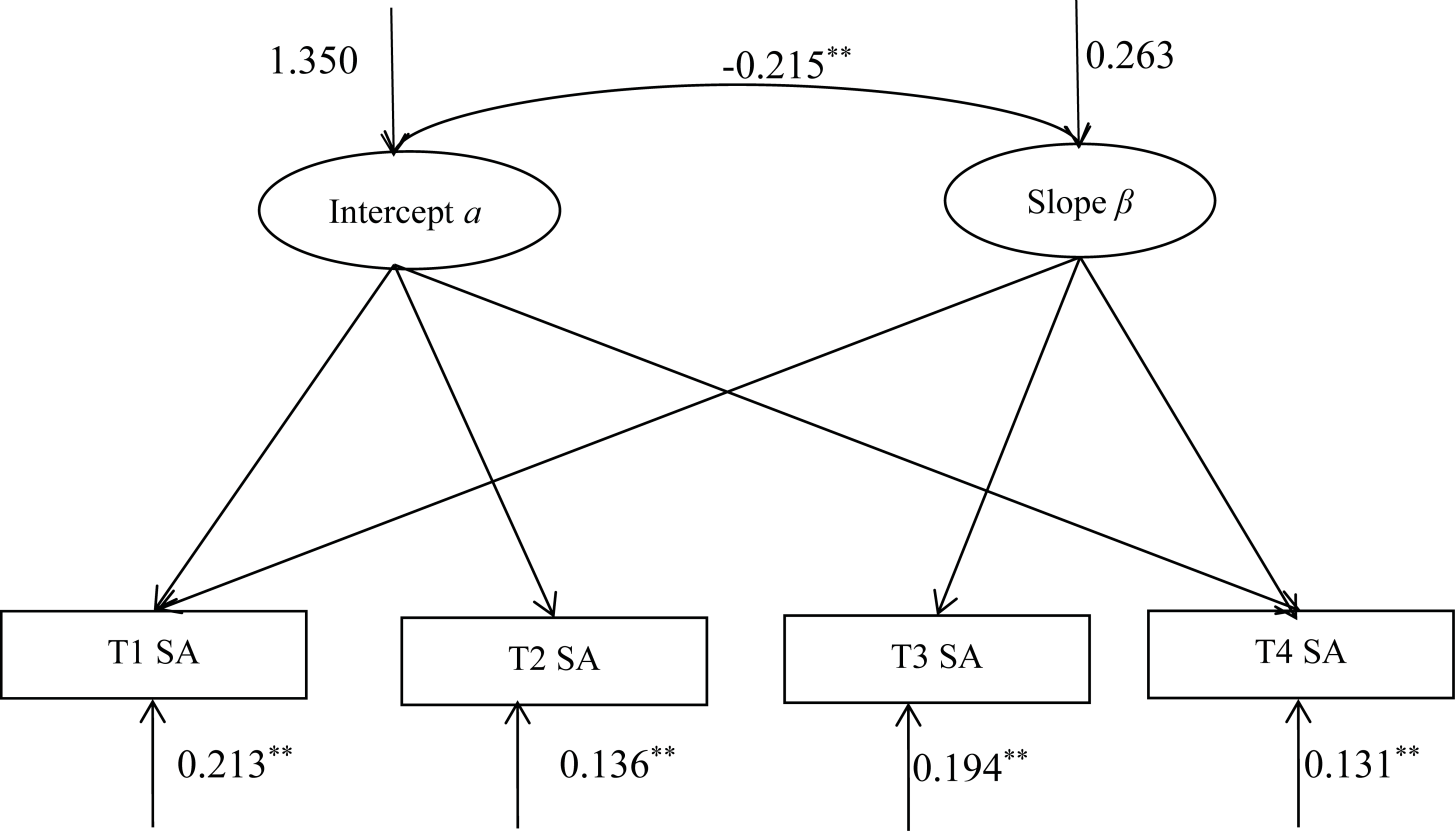
Linear developmental trajectories of adolescent social anxiety. All parameters in the figure are standardized path coefficients, ** indicates *P* < 0.01, and this notation will be used throughout.

To investigate the developmental trajectories of adolescent social anxiety, this study utilized latent growth curve modeling. As depicted in Figure 4, the factor loadings for social anxiety across the four time points all exceeded 0.67 and were statistically significant (*p* < 0.01), indicating that the observed indicators contributed meaningfully to the latent construct. Model fit was assessed based on standard fit indices (e.g., CFI, TLI, RMSEA, and SRMR), not solely on factor loadings, and the fit indices suggested an acceptable fit.

The study proceeded to construct both linear and nonlinear unconditional latent growth models for social anxiety to further elucidate the specific characteristics of its developmental trajectory. As presented in Table 3, the linear unconditional model exhibited satisfactory fit indices (χ^2^(10) = 11.028, *p* = 0.012, CFI = 0.986, RMSEA = 0.012, SRMR = 0.028), indicating a good fit to the data. The model outcomes revealed that the initial level of social anxiety (i.e., the intercept) was 0.716, which was statistically significant but did not vary significantly among individuals (1.350, *p* > 0.05). The change rate of social anxiety (i.e., the slope) was 0.035, which was not statistically significant and showed no significant variation among individuals (0.263, *p* > 0.05). This suggests that the overall level of social anxiety among adolescents remained relatively stable, with minimal variation in the rate of change across individuals.

**TABLE 3:** Coefficients and fit indices for linear and nonlinear unconditional latent growth models of social anxiety.

Model	*x^2^ (df)*	*P*	*CFI*	*RMSEA*	*SRMR*	Coefficient			Variance		
						Intercept	Slope	Nonlinear slope	Intercept	Slope	Nonlinear slope
Linear unconditional model	11.028 (10)	0.012	0.986	0.012	0.028	0.716**	0.035	–	1.350	0.263	–
Nonlinear unconditional model	16.887 (13)	0.001	0.995	0.014	0.002	2.713**	0.178**	−0.064**	1.581**	0.889**	0.062

*indicates *P* < 0.05; **indicates *P* < 0.01; ***indicates *P* < 0.001.

**FIGURE 5:**
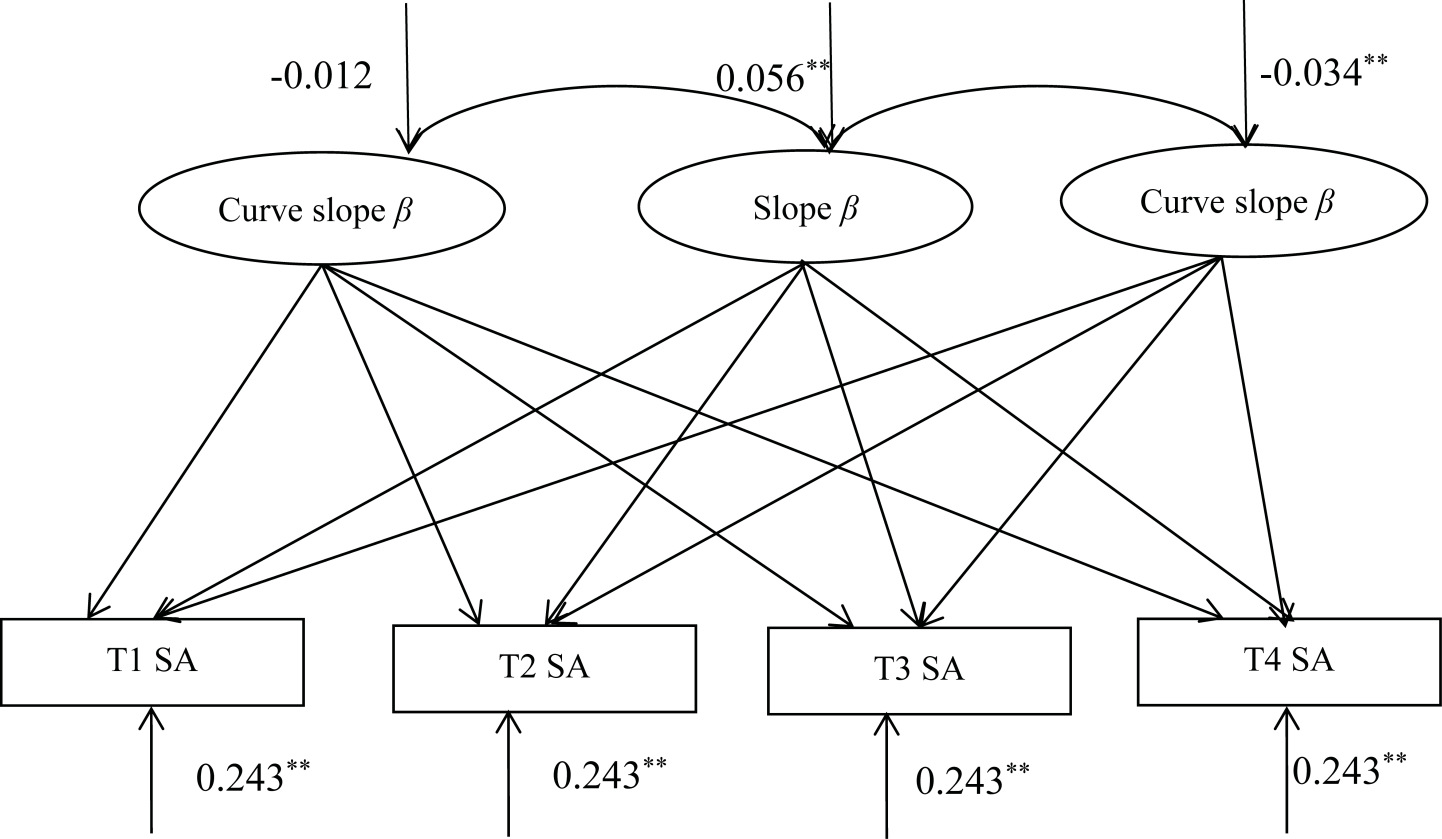
Nonlinear developmental trajectories of adolescent social anxiety. All parameters in the figure are standardized path coefficients, ** indicates *P* < 0.01, and this applies to subsequent figures as well.

The nonlinear unconditional model also demonstrated good fit indices (χ^2^(13) = 16.887, *p* = 0.001, CFI = 0.995, RMSEA = 0.014, SRMR = 0.002), as illustrated in Figure 5. The model results indicated that the initial level of social anxiety was 2.713, which was statistically significant and varied significantly among individuals (1.581, *p* < 0.01). The linear change rate of social anxiety (i.e., the slope) was 0.178, which was statistically significant and showed significant variation among individuals (0.889, *p* < 0.01). The nonlinear change rate of social anxiety (i.e., the curvature slope) was −0.064, which was statistically significant but did not significantly vary among individuals (0.062, *p* > 0.05). This indicates that the developmental trajectory of adolescent social anxiety has significant nonlinear characteristics, exhibiting a trend of initial increase followed by a decrease, with significant differences in the initial level and linear change rate among individuals, but no significant variation in the nonlinear change rate.

### 3.4 Multivariate developmental trajectories of adolescent physical activity and social anxiety

**FIGURE 6:**
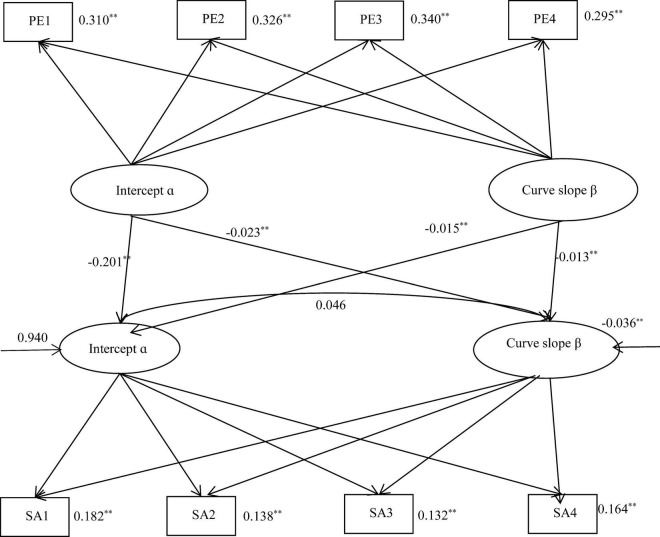
Multivariate developmental trajectories of adolescent physical activity and social anxiety. All parameters in the figure are standardized path coefficients, ** indicates *P* < 0.01, and this notation will be used throughout subsequent figures.

To further explore the multivariate developmental trajectories of adolescent physical activity and social anxiety, this study constructed a multivariate latent growth model based on the previous analyses. As shown in Figure 6, the factor loadings for both physical activity and social anxiety at the four time points were all above 0.52 and reached significant levels (*p* < 0.01), indicating that the measurement model fits the data well and that the measurements of physical activity and social anxiety at the four time points can effectively reflect their latent variable characteristics.

According to Table 4, the fit indices of the multivariate latent growth model are good (χ^2^(15) = 20.500, CFI = 0.990, TLI = 0.985, RMSEA = 0.020, SRMR = 0.018), suggesting that the model fits the data well. From the key parameter estimates in Table 5, the initial level of physical activity (i.e., the intercept) has a significant negative predictive effect on the initial level of social anxiety (β = −0.250, *p* < 0.001), indicating that higher initial levels of physical activity are associated with lower initial levels of social anxiety; the initial level of physical activity also has a significant negative predictive effect on the change rate of social anxiety (i.e., the slope) (β = −0.154, *p* < 0.001), indicating that higher initial levels of physical activity are associated with slower developmental rates of social anxiety; the change rate of physical activity also has a significant negative predictive effect on the change rate of social anxiety (β = −0.102, *p* < 0.001), indicating that faster developmental rates of physical activity are associated with slower developmental rates of social anxiety.

**TABLE 4:** Fit indices for the multivariate latent growth curve modeling (lgcm) of adolescent physical activity and social anxiety.

χ^2^	df	CFI	TLI	RMSEA	SRMR
20.500	15	0.990	0.985	0.020	0.018

**TABLE 5:** Key parameter estimates of the multivariate latent growth curve modeling (LGCM) for adolescent physical activity and social anxiety.

Path	Standardized coefficient	SE	*P*-value
Physical activity intercept → social anxiety intercept	−0.250	0.012	<0.001
Physical activity intercept → social anxiety slope	−0.154	0.007	<0.001
Physical activity slope → social anxiety slope	−0.102	0.003	<0.001

## 4 Discussion

### 4.1 Trajectories of adolescent physical activity

This study used latent growth curve modeling to examine the developmental trajectories of adolescent physical activity, revealing that adolescent physical activity levels generally exhibit stability with slight variability. Specifically, the initial level was high with significant individual differences, and the developmental trend showed a slight decline accompanied by certain nonlinear characteristics.

The analysis of the linear unconditional latent growth model indicated that the initial level of adolescent physical activity was significant, with significant individual differences, suggesting that there are clear individual differences in the initial stage of physical activity among adolescents. This result is consistent with existing research that suggests adolescent sports participation is influenced by various factors, such as family support, school environment, and personal interests (Graham et al., 2011; Sharifi et al., 2024; Wang J. et al., 2024). Additionally, the significant change rate suggests an overall slight downward trend in adolescent physical activity levels, which may be related to increased academic pressure and reduced social activity time among adolescents (Eime et al., 2013; Kemp et al., 2022; Latha, 2024; Wang et al., 2021).

The nonlinear unconditional latent growth model further revealed the nonlinear characteristics of physical activity, with significant nonlinear change rates indicating a “decreasing then increasing” trend in the developmental trajectory of adolescent physical activity. This nonlinear pattern may reflect time-varying fluctuations in physical exercise within the school year; potential contextual explanations (e.g., academic workload or time allocation) were not measured in the present study and are therefore not further interpreted here (Campos et al., 2024; Haverkamp et al., 2020; Ishihara et al., 2020; Qi et al., 2024; Sasayama et al., 2024; Wang and Eccles, 2012).

The model results also showed that there were individual differences in the change rate of physical activity, but no significant individual differences in the nonlinear change rate. This may reflect that some adolescents, due to differences in personal interests, family environment, or school support, show more pronounced changes in physical exercise participation; however, in the present four-wave data, the nonlinear (quadratic) change did not significantly vary across individuals, suggesting limited between-person differences in the curvature component (Allen et al., 2021; McGaughey et al., 2020; Rinta-Antila et al., 2023; Wang J. et al., 2024).

In summary, this study reveals that adolescent physical activity trajectories exhibit notable linear and nonlinear patterns alongside significant individual variability (Fu et al., 2024; McDonough and Knight, 2024; O’Donnell et al., 2024; Wang J. et al., 2024; Yang, 2024). These insights underscore the critical role of physical activity in adolescent development and highlight the need for targeted interventions that consider developmental stages and individual traits, such as fostering conducive family and school settings to encourage ongoing physical engagement.

### 4.2 Trajectories of adolescent social anxiety

This study examined the developmental trajectories of adolescent social anxiety, revealing significant nonlinear changes in the levels of social anxiety among adolescents, with substantial individual differences observed in both initial levels and rates of development.

Linear unconditional latent growth model analysis showed that the initial level of adolescent social anxiety was statistically significant, but individual differences were not significant, meaning that most adolescents had relatively consistent levels of social anxiety at the beginning of the study. This may be related to the common social challenges faced by adolescents in the early stage, such as the need for peer acceptance and concerns about self-image (Gómez-Ortiz et al., 2018; Wu et al., 2021; Shang et al., 2023). However, the change rate of social anxiety was not significant overall, and individual differences were also not significant, indicating that the level of social anxiety among adolescents remained relatively stable during the study period, which is consistent with some literature suggesting that social anxiety in adolescence is stably influenced by personality traits and environmental factors (Chubar et al., 2020; Goldsmith et al., 2022; Lemyre et al., 2019; Rapee et al., 2022; Wang F. et al., 2024).

The nonlinear unconditional latent growth model further revealed the nonlinear development characteristics of adolescent social anxiety, with model results indicating a “rising then falling” trend in the developmental trajectory of social anxiety. This pattern may be related to time-varying changes in adolescents’ social anxiety during the study period; specific explanatory factors (e.g., peer competition, academic pressure, or coping strategies) were not directly measured and are therefore discussed only as possible interpretations (Chen L. M. et al., 2024; Hellfeldt et al., 2020; Kahi et al., 2024; Korja et al., 2024; La Greca and Harrison, 2005). There were significant individual differences in the initial level and linear change rate of social anxiety, indicating that some adolescents may exhibit individualized characteristics in the development of social anxiety due to differences in personality, family support, or social skills (Chen et al., 2025; Huttunen et al., 2024; Tehrani et al., 2024; Zhou et al., 2024).

The individual differences in nonlinear changes were not significant, which may mean that although adolescents generally experienced a similar developmental pattern in anxiety levels, individualized interventions and support did not significantly change their developmental trajectories. This finding suggests that intervention measures may need to be more refined and personalized to provide effective support for different social situations and psychological characteristics (Cougle et al., 2020; Pelissolo et al., 2019; Piccirillo and Rodebaugh, 2022).

Theoretically, the trajectory of social anxiety in adolescents reflects a dynamic psychological adaptation process. Linked to self-esteem (Chen and Qin, 2020; Kong et al., 2022; Wu et al., 2021), social anxiety has been discussed in the literature in relation to family and social support (Aune et al., 2021; Kelly and Malecki, 2022; Mo and Bai, 2023; Spence et al., 2022); however, these constructs were not directly measured in the present study and are therefore not interpreted as mechanisms here. Adolescents perceiving less social support tend to feel more isolated, increasing their anxiety (Chi et al., 2023; Kelly and Malecki, 2022; Spence et al., 2022). However, support from family and peers can over time improve emotional regulation and reduce social anxiety (Halidu and Kotera, 2024; King et al., 2024; Musella et al., 2023; Zhou et al., 2024).

In conclusion, this study highlights the nonlinear development of adolescent social anxiety and inter-individual differences. These findings suggest that interventions may consider adolescents’ different needs across developmental stages. Although emotional regulation and social support were not examined in the present analyses, they may be relevant targets for future mechanism-focused research and intervention design.

### 4.3 The impact of adolescent physical activity on the developmental trajectories of social anxiety

This study used multivariate latent growth curve modeling to examine the dynamic association between adolescent physical activity and social anxiety. The results showed that physical activity was negatively associated with social anxiety over time. The findings showed that higher initial levels of physical activity were associated with lower initial levels of social anxiety. This is consistent with the proposed pathway that physical activity may be associated with social anxiety through neurobiological and psychological mechanisms. Specifically, physical activity (including exercise) may help regulate stress-related physiological responses and neurotransmitter activity, which may further improve self-efficacy, emotion regulation, and social competence (Banai Tizkar et al., 2024; Darajat and Nuryadi, 2017; Faizan et al., 2024; Fathirezaie et al., 2024; Li S. et al., 2024; Nazari et al., 2020; Sartika et al., 2023; Zheng and Ye, 2022).

The study further established that the initial level of physical activity negatively predicts the rate of social anxiety development, suggesting that physically active adolescents experience a slower increase in social anxiety (Guo and Liang, 2023; Huang, 2024; Ma et al., 2024). This suggests that physical exercise may be associated with slower increases in social anxiety over time; because potential mediating mechanisms (e.g., teamwork, coping, or social confidence) were not measured, we do not draw mechanism-based conclusions from the present data (Li Q. et al., 2024; Liu et al., 2023; Sheng et al., 2024).

Moreover, a faster developmental rate of physical activity was found to negatively predict the rate of social anxiety increase, indicating that adolescents who increase their physical activity quickly are less likely to see a sharp rise in social anxiety. This finding highlights the potential value of physical exercise as a potential intervention target and suggests that promoting exercise among adolescents may be associated with slower increases in social anxiety over time (Belcher et al., 2021; Ji et al., 2024). Theoretically, these findings also support the evolutionary psychology view that regular movement and social interaction may help reduce the mismatch between adolescents’ evolved needs and modern sedentary contexts. Moreover, these findings are consistent with Social Learning Theory, Cognitive Theory, the Stress Buffering Model, and Self-Determination Theory. From a Self-Determination Theory perspective, engagement in physical exercise may support basic psychological needs (e.g., competence, relatedness, and autonomy), which may in turn be linked to better emotional adjustment and lower social anxiety. From the perspective of Social Learning Theory, exercise settings may allow adolescents to learn social behaviors through observation, imitation, and peer interaction. Cognitive Theory suggests that successful participation in exercise may improve self-efficacy and reduce negative self-evaluation. The Stress Buffering Model indicates that exercise-related social support and coping resources may weaken the impact of stress on social anxiety. Self-Determination Theory further suggests that exercise may satisfy adolescents’ needs for competence and relatedness, thereby improving psychological resilience and social adaptation (Bushnell, 2024; Li et al., 2023; Panza et al., 2020; Wang, 2024).

However, the efficacy of physical activity on social anxiety may be modulated by individual traits and environmental factors. For example, adolescents with lower self-esteem may require more sustained or structured exercise programs to obtain emotional benefits (Dale et al., 2019; Zhang Y. et al., 2024). Family and school support also significantly influence the positive effects of physical activity (Lifeng, 2022). Future interventions should, therefore, integrate multi-level support, creating opportunities for exercise and providing emotional and social support to better adapt adolescents to social situations.

In conclusion, this study identified a close association between physical exercise and social anxiety in adolescents, suggesting that exercise co-occurs with lower levels of social anxiety and may support mental health. These insights provide hypothesis-generating evidence that may inform future research on adolescent mental health, including the design of theory-driven and, ideally, experimental or intervention studies to test whether increasing physical exercise is associated with changes in social anxiety. Future research should further examine the specific effects of different exercise types, intensities, and frequencies, which were not analyzed separately in the present study, as well as the synergistic role of family and school support, to inform personalized and targeted intervention plans.

### 4.4 Limitations

Firstly, this study employed latent growth curve modeling to examine the developmental trajectories of general physical activity participation and social anxiety in adolescents; however, it did not separately analyze the effects of specific exercise types, intensities, or frequencies. In addition, this study did not fully explore contextual factors such as family economic status, school support, and peer pressure. Future research should include more detailed physical activity indicators and contextual factors to provide a more comprehensive understanding of adolescent mental health. In addition, the baseline sample was male-skewed (62.51% male), which may limit generalizability; future studies may consider recruiting more gender-balanced samples and/or conducting multi-group analyses to further examine potential gender differences. Secondly, relying on self-reports from adolescents, this study’s data collection might be prone to social desirability or memory biases, which could skew the actual levels of physical activity and social anxiety. Future studies could use multi-source data, including reports from parents and teachers, to improve the reliability of the findings. Third, although the four-wave longitudinal design reduced some concerns related to missing data, sample attrition may still have influenced the findings. Therefore, future research should aim for longer-term tracking and higher retention rates to further validate the current findings. In addition, because this study was observational, the findings should be interpreted as longitudinal associations rather than causal effects. Several potentially important confounders (e.g., socioeconomic status, diet, sleep, and family/peer context) were not measured, which may have introduced residual confounding. Finally, students were sampled within schools; future studies may consider multilevel/cluster-robust approaches to better account for school-level clustering and strengthen generalizability.

## 5 Conclusion

This study examined the developmental trajectories of physical activity and social anxiety in adolescents and their dynamic association using latent growth curve modeling, revealing the following key conclusions: (1) The initial level and rate of change in physical exercise negatively predicted the development of social anxiety, suggesting that physical exercise co-occurs with lower social anxiety and slower increases in social anxiety over time. (2) The developmental trajectory of social anxiety shows significant nonlinear characteristics, with a “rising then falling” pattern, and significant individual differences in initial levels and rates of change, reflecting the uniqueness and dynamics of adolescents in dealing with complex social situations. (3) Physical exercise may serve as an important protective factor for mental health and may provide a basis for interventions aimed at reducing adolescent social anxiety and improving psychological adaptation. Potential mechanisms (e.g., emotional regulation and social support) were not directly measured or tested in the present study and therefore remain speculative.

## Data Availability

The datasets presented in this study can be found in online repositories. The names of the repository/repositories and accession number(s) can be found in the article/supplementary material.

## References

[B1] Allen M. S. Robson D. A. Vella S. A. Laborde S. (2021). Extraversion development in childhood, adolescence and adulthood: Testing the role of sport participation in three nationally-representative samples. *J. Sports Sci.* 39 2258–2265. 10.1080/02640414.2021.1930672 34013834

[B2] Aune T. Juul E. M. Beidel D. C. Nordahl H. Dvorak R. (2021). Mitigating adolescent social anxiety symptoms: The effects of social support and social self-efficacy in findings from the Young-HUNT 3 study. *Eur. Child Adolesc. Psychiatry* 30 441–449. 10.1007/s00787-020-01529-0 32300894 PMC8019414

[B3] Bae M. H. Zhang X. Lee J. S. (2024). Exercise, grit, and life satisfaction among Korean adolescents: A latent growth modeling analysis. *BMC Public Health* 24:1392. 10.1186/s12889-024-18899-8 38783255 PMC11119792

[B4] Banai Tizkar R. McIver L. Wood C. M. (2024). Subcallosal area 25: Its responsivity to the stress hormone cortisol and its opposing effects on appetitive motivation in marmosets. *Neurobiol. Stress* 31:100637. 10.1016/j.ynstr.2024.100637 38741617 PMC11089406

[B5] Belcher B. R. Zink J. Azad A. Campbell C. Chakravartti S. Herting M. et al. (2021). The roles of physical activity, exercise, and fitness in promoting resilience during adolescence: Effects on mental well-being and brain development. *Biol. Psychiatry Cogn. Neurosci. Neuroimaging* 6 225–237. 10.1016/j.bpsc.2020.08.005 33067166 PMC7878276

[B6] Bushnell A. (2024). *The Role of Team Sports, Coping, and Friendship in Reducing Depressive Symptoms in Youth. College of Science and Health Theses and Dissertations.* Chicago, IL: DePaul University.

[B7] Campos N. M. Medeiros M. Neto P. F. (2024). The impact of sports participation on cognitive functions and academic performance among youth aged 10 to 14 years: A comprehensive investigation. *Res. Soc. Dev.* 13:e8213244697. 10.33448/rsd-v13i2.44697

[B8] Cécillon F. X. Mermillod M. Leys C. Bastin H. Lachaux J. Shankland R. (2024). The reflective mind of the anxious in action: Metacognitive beliefs and maladaptive emotional regulation strategies constrain working memory efficiency. *Eur. J. Invest. Health Psychol. Educ.* 14 505–530. 10.3390/ejihpe14030034 38534895 PMC10968987

[B9] Chen C. Qin J. (2020). Emotional abuse and adolescents’ social anxiety: The roles of self-esteem and loneliness. *J. Fam. Viol.* 35 497–507. 10.1007/s10896-019-00099-3

[B10] Chen L. M. Pokhvisneva I. Lahti-Pulkkinen M. Kvist T. Baldwin J. Parent C. et al. (2024). Independent prediction of child psychiatric symptoms by maternal mental health and child polygenic risk scores. *J. Am. Acad. Child Adolesc. Psychiatry* 63 640–651. 10.1016/j.jaac.2023.08.018 37977417 PMC11105503

[B11] Chen L. Chen F. Bai S. Xiu Z. Ding Y. (2025). Social-emotional competence among adolescents: A network approach to model relationships among social-emotional competence, emotion regulation, and anxiety. *Pers. Individ. Dif.* 236:113023. 10.1016/j.paid.2024.113023

[B12] Chen S. Jing L. Li C. Wang H. (2024). Exploring the nexus between moderate-to-vigorous physical activity, self-disclosure, social anxiety, and adolescent social avoidance: Insights from a cross-sectional study in central China. *Children* 11:56. 10.3390/children11010056 38255369 PMC10814873

[B13] Chi X. Jiang W. Guo T. (2023). Relationship between adverse childhood experiences and anxiety symptoms among Chinese adolescents: The role of self-compassion and social support. *Curr. Psychol.* 42 12822–12834. 10.1007/s12144-021-02534-5 35035184 PMC8741560

[B14] Chubar V. Leeuwen K. V. Bijttebier P. Van Assche E. Bosmans G. Van den Noortgate W. et al. (2020). Gene–environment interaction: New insights into perceived parenting and social anxiety among adolescents. *Eur. Psychiatry* 63:e64. 10.1192/j.eurpsy.2020.62 32507125 PMC7355173

[B15] Cougle J. R. Mueller N. E. McDermott K. A. Wilver N. L. Carlton C. N. Okey S. A. (2020). Text message safety behavior reduction for social anxiety: A randomized controlled trial. *J. Consult. Clin. Psychol.* 88, 445–454. 10.1037/ccp0000477 32105093

[B16] Dale L. P. Vanderloo L. Moore S. Faulkner G. (2019). Physical activity and depression, anxiety, and self-esteem in children and youth: An umbrella systematic review. *Mental Health Phys. Activity* 16 66–79. 10.1016/j.mhpa.2018.12.001

[B17] Darajat J. K. Nuryadi G. A. (2017). The effect of physical fitness and healthy behavior toward concentration, anxiety and cortisol hormone. *IOP Conf. Ser. Mater. Sci. Eng.* 180:12208. 10.1088/1757-899X/180/1/012208

[B18] Deng Y. Wang X. (2024). The impact of physical activity on social anxiety among college students: The chain mediating effect of social support and psychological capital. *Front. Psychol.* 15:1406452. 10.3389/fpsyg.2024.1406452 38957885 PMC11217649

[B19] Duncan S. C. Duncan T. E. Biglan A. Ary D. (1998). Contributions of the social context to the development of adolescent substance use: A multivariate latent growth modeling approach. *Drug Alcohol Dependence* 50 57–71. 10.1016/s0376-8716(98)00006-4 9589273

[B20] Duncan T. E. Duncan S. C. Strycker L. A. (2013). *An Introduction to Latent Variable Growth Curve Modeling: Concepts, Issues, and Application*, 2nd Edn. New York: Routledge, 274.

[B21] Eime R. M. Harvey J. T. Sawyer N. A. Craike M. Symons C. Polman R. et al. (2013). Understanding the contexts of adolescent female participation in sport and physical activity. *Res. Quart. Exerc. Sport* 84 157–166. 10.1080/02701367.2013.784846 23930541

[B22] Faizan M. Ahuja A. Ansari S. Sharma S. (2024). Effect of 6-week combined training on salivary cortisol levels, total Olympic lifting performance and anxiety levels in elite male Olympic weightlifters. *Sport Sci. Health* 20 55–64. 10.1007/s11332-023-01064-w

[B23] Fang S. Mushtaque I. (2024). The moderating role of health literacy and health promoting behavior in the relationship among health anxiety, emotional regulation, and cyberchondria. *Psychol. Res. Behav. Manag.* 17 51–62. 10.2147/PRBM.S446448 38196775 PMC10775698

[B24] Fathirezaie Z. Badicu G. Yagin F. H. Aghdasi M. Sani S. Z. Abbaspour K. et al. (2024). Personality and motivation of physical activity in adolescent girls: Effects of perceived parental support and social physical anxiety. *BMC Public Health* 24:799. 10.1186/s12889-024-18060-5 38481212 PMC10938660

[B25] Flick L. Dawes M. Brian A. (2024). Relationships among peer- relatedness, self-confidence, peer victimization, social anxiety and school satisfaction in American high school students. *Phys. Educ. Sport Pedagogy* 29 573–587. 10.1080/17408989.2022.2135692

[B26] Fredrick J. W. Luebbe A. M. (2024). Prospective associations between fears of negative evaluation, fears of positive evaluation, and social anxiety symptoms in adolescence. *Child Psychiatry Hum. Dev.* 55 195–205. 10.1007/s10578-022-01396-7 35790648 PMC9255539

[B27] Fu L. Burns R. D. Zhe S. Bai Y. (2024). What explains adolescents’ physical activity and sports participation during the COVID-19 pandemic? – An interpretable machine learning approach. *J. Sports Sci.* 42 1651–1663. 10.1080/02640414.2024.2404783 39300762

[B28] Gaudlitz K. von Lindenberger B. L. Zschucke E. Ströhle A. (2015). “Mechanisms underlying the relationship between physical activity and anxiety: Human data,” in *Routledge Handbook of Physical Activity and Mental Health*, ed. Ekkekakis P. (Milton Park: Routledge).

[B29] Gautam N. Rahman M. M. Khanam R. (2024). Adverse childhood experiences and externalizing, internalizing, and prosocial behaviors in children and adolescents: A longitudinal study. *J. Affect. Disord.* 363 124–133. 10.1016/j.jad.2024.07.064 39043305

[B30] Goldsmith H. H. Hilton E. C. Phan J. M. Sarkisian K. Carroll I. Lemery-Chalfant K. et al. (2022). Childhood inhibition predicts adolescent social anxiety: Findings from a longitudinal twin study. *Dev. Psychopathol.* 34 1666–1685. 10.1017/S0954579422000864 36229958 PMC10102261

[B31] Gómez-Ortiz O. Roldán R. Ortega-Ruiz R. (2018). Social anxiety and psychosocial adjustment in adolescents: Relation with peer victimization, self-esteem and emotion regulation. *Child Ind. Res.* 11 1719–1736. 10.1007/s12187-017-9506-3

[B32] Graham D. J. Schneider M. Dickerson S. S. (2011). Environmental resources moderate the relationship between social support and school sports participation among adolescents: A cross-sectional analysis. *Int. J. Behav. Nutr. Phys. Act* 8:34. 10.1186/1479-5868-8-34 21501504 PMC3103410

[B33] Gunzler D. D. Morris N. (2015). A tutorial on structural equation modeling for analysis of overlapping symptoms in co-occurring conditions using MPlus. *Stat. Med.* 34 3246–3280. 10.1002/sim.6541 26045102 PMC4592386

[B34] Guo L. Liang L. (2023). Physical activity as a causal variable for adolescent resilience levels: A cross-lagged analysis. *Front. Psychol.* 14:1095999. 10.3389/fpsyg.2023.1095999 36910759 PMC9992974

[B35] Halidu M. Kotera Y. (2024). Adolescent social anxiety, school satisfaction, family emotional support, and school absenteeism: Findings from young-HUNT3 and Norwegian national education data. *J. Clin. Med.* 13:2547. 10.3390/jcm13092547 38731079 PMC11084760

[B36] Haverkamp B. F. Wiersma R. Vertessen K. van Ewijk H. Oosterlaan J. Hartman E. (2020). Effects of physical activity interventions on cognitive outcomes and academic performance in adolescents and young adults: A meta-analysis. *J. Sports Sci.* 38 2637–2660. 10.1080/02640414.2020.1794763 32783695

[B37] Hayatipoor S. Bavi S. Khalafi A. Dasht Bozorgi Z. Gatezadeh A. (2024). Effects of stress management training on cognitive avoidance and emotion regulation strategies in female students with social anxiety disorder: A mindfulness and emotional schema therapy approach. *Int. J. School Health* 11 40–49. 10.30476/INTJSH.2023.100698.1357 PMC13325663 42519132

[B38] Hellfeldt K. López-Romero L. Andershed H. (2020). Cyberbullying and psychological well-being in young adolescence: The potential protective mediation effects of social support from family, friends, and teachers. *Int. J. Environ. Res. Public Health* 17:45. 10.1155/2024/6662382 PMC6981789 31861641

[B39] Hossain M. N. Lee J. Choi H. Kwak Y. Kim J. (2024). The impact of exercise on depression: How moving makes your brain and body feel better. *Phys. Act Nutr.* 28 43–51. 10.20463/pan.2024.0015 39097997 PMC11298280

[B40] Huang D. (2024). Research on the relationship between school physical education and students’ comprehensive development. *Int. J. Educ. Teach. Res.* 1:307. 10.70767/ijetr.v1i2.307

[B41] Huttunen I. Upadyaya K. Salmela-Aro K. (2024). Longitudinal associations between adolescents’ social-emotional skills, school engagement, and school burnout. *Learn. Individ. Dif.* 115:102537. 10.1016/j.lindif.2024.102537

[B42] Ishihara T. Nakajima T. Yamatsu K. Okita K. Sagawa M. Morita N. (2020). Relationship of participation in specific sports to academic performance in adolescents: A 2-year longitudinal study. *Scand. J. Med. Sci. Sports* 30 1471–1482. 10.1111/sms.13703 32350922

[B43] Jetten J. Haslam C. von Hippel C. Bentley S. Cruwys T. Steffens N. et al. (2022). “Let’s get physical” — or social: The role of physical activity versus social group memberships in predicting depression and anxiety over time. *J. Affect. Disord.* 306 55–61. 10.1016/j.jad.2022.03.027 35301039

[B44] Ji F. Sun Q. Han W. Li Y. Xia X. (2024). How physical exercise reduces problematic mobile phone use in adolescents: The roles of expression suppression, depression, anxiety, and resilience. *Psychol. Res. Behav. Manag.* 17 4369–4382. 10.2147/PRBM.S484089 39722776 PMC11669333

[B45] Kahi M. Heidari Z. Esmaeili M. (2024). From screens to minds: The connection between cyberbullying, psychological well-being, and the mediating role of perceived social support in high school students. *Adv. Public Health* 2024:6662382. 10.1155/2024/6662382

[B46] Kajastus K. Haravuori H. Kiviruusu O. Marttunen M. Ranta K. (2024). Associations of generalized anxiety and social anxiety with perceived difficulties in school in the adolescent general population. *J. Adolesc.* 96 291–304. 10.1002/jad.12275 37985185

[B47] Kelly K. M. Malecki C. K. (2022). Social Support, depression, and anxiety in female adolescents: Associations and profiles. *Child Youth Care Forum* 51 85–109. 10.1007/s10566-021-09617-1

[B48] Kemp B. J. Cliff D. P. Kariippanon K. E. Crowe R. Parrish A. (2022). ‘Not just for fun anymore’: A qualitative exploration of social norms related to the decline in non-organised physical activity between childhood and adolescence in Australia. *Sport Educ. Soc.* 27 41–56. 10.1080/13573322.2020.1822795

[B49] Kim C. Kim J. Thapa B. (2021). Intensity of leisure-time physical activity and dimensions of mental well-being: A reciprocal approach using parallel latent growth curve modeling. *J. Phys. Act Health* 18 165–174. 10.1123/jpah.2020-0300 33429358

[B50] King E. K. Halbreich E. D. Callina K. Mueller M. K. (2024). Companion animals and the relationship between peer victimization and emotion regulation in youth. *J. Res. Adolesc.* 34 85–95. 10.1111/jora.12901 37975498

[B51] Kong F. Lan N. Zhang H. (2022). How does social anxiety affect Mobile phone dependence in adolescents? The mediating role of self-concept clarity and self-esteem. *Curr. Psychol.* 41 8070–8077. 10.1007/s12144-020-01262-6

[B52] Korja R. Nolvi S. Scheinin N. M. Tervahartiala K. Carter A. Karlsson H. et al. (2024). Trajectories of maternal depressive and anxiety symptoms and child’s socio-emotional outcome during early childhood. *J. Affect. Disord.* 349 625–634. 10.1016/j.jad.2023.12.076 38184113

[B53] La Greca A. M. Harrison H. M. (2005). Adolescent peer relations, friendships, and romantic relationships: Do they predict social anxiety and depression? *J. Clin. Child Adolesc. Psychol.* 34 49–61. 10.1207/s15374424jccp3401_5 15677280

[B54] Latha B. (2024). Impact of parental pressure and involvement on youth sports participation: A study. *Library Prog. Int.* 44 11612–11622.

[B55] Lemyre A. Gauthier-Légaré A. Bélanger R. E. (2019). Shyness, social anxiety, social anxiety disorder, and substance use among normative adolescent populations: A systematic review. *Am. J. Drug Alcohol Abuse* 45 230–247. 10.1080/00952990.2018.1536882 30422012

[B56] Li J. Xue E. Liu C. (2023). Integrated macro and micro analyses of student burden reduction policies in China: Call for a collaborative “family–school–society” model. *Humanit. Soc. Sci. Commun.* 10 1–10. 10.1057/s41599-023-01695-x

[B57] Li Q. Li L. Li C. Wang H. (2024). The association between moderate-to-vigorous physical activity and health-related quality of life in Chinese adolescents: the mediating roles of emotional intelligence and perceived stress. *Front. Psychol.* 15:1477018. 10.3389/fpsyg.2024.1477018 39687563 PMC11646723

[B58] Li S. Luo X. Song L. Gao X. Shen Y. (2024). Childhood maltreatment and adolescent eating disorders’ symptoms: A moderated mediation model of social anxiety and physical activity. *Psychol. Res. Behav. Manag.* 17 3875–3887. 10.2147/PRBM.S489186 39559712 PMC11570528

[B59] Li X. Zhu Y. Shi X. (2024). Interpersonal sensitivity as a mediator linking interpersonal stressors and social anxiety: Longitudinal mediation analysis using parallel process latent growth curve modeling. *J. Affect. Disord.* 351 172–178. 10.1016/j.jad.2024.01.218 38296055

[B60] Lifeng X. (2022). A home-based sports training in physical health promotion for university students. *Rev. Bras. Med. Esporte* 29:e2022_0220. 10.1590/1517-8692202329012022_0220

[B61] Liu Y. Chen J. Chen K. Liu J. Wang W. (2023). The associations between academic stress and depression among college students: A moderated chain mediation model of negative affect, sleep quality, and social support. *Acta Psychol.* 239:104014. 10.1016/j.actpsy.2023.104014 37633175

[B62] Ma H. Y. Chiang N. T. Kao R. H. Lee C. (2024). Health Workers’ mindfulness-based stress reduction and resilience during COVID-19 pandemic. *J. Multidisciplinary Healthc.* 17 3691–3713. 10.2147/JMDH.S464285 39114858 PMC11303674

[B63] Marcoulides K. M. Yuan K. H. (2017). New ways to evaluate goodness of fit: A note on using equivalence testing to assess structural equation models. *Struct. Equ. Model. Multidisciplinary J.* 24 148–153. 10.1080/10705511.2016.1225260

[B64] McDonough K. Knight E. K. (2024). The role of sport in juvenile justice: Benefits according to key informants. *Sport Soc.* 27 1603–1623. 10.1080/17430437.2024.2307496

[B65] McGaughey T. Vlaar J. Naylor P. J. Hanning R. Le Mare L. Mâsse L. (2020). Individual and environmental factors associated with participation in physical activity as adolescents transition to secondary school: A qualitative inquiry. *Int. J. Environ. Res. Public Health* 17:7646. 10.3390/ijerph17207646 33092157 PMC7588993

[B66] Meyerhoff J. Liu T. Kording K. P. Ungar L. Kaiser S. Karr C. et al. (2021). Evaluation of changes in depression, anxiety, and social anxiety using smartphone sensor features: Longitudinal cohort study. *J. Med. Internet Res.* 23:e22844. 10.2196/22844 34477562 PMC8449302

[B67] Mo Q. Bai B. (2023). Height dissatisfaction and loneliness among adolescents: The chain mediating role of social anxiety and social support. *Curr. Psychol.* 42 27296–27304. 10.1007/s12144-022-03855-9 36277262 PMC9579572

[B68] Mou Q. Zhuang J. Wu Q. Zhong Y. Dai Q. Cao X. et al. (2024). Social media addiction and academic engagement as serial mediators between social anxiety and academic performance among college students. *BMC Psychol.* 12:190. 10.1186/s40359-024-01635-7 38582933 PMC10998323

[B69] Mu F. Liu J. Lou H. Zhu W. Wang Z. Li B. et al. (2024). How breaking a sweat affects mood: The mediating role of self-efficacy between physical exercise and emotion regulation ability. *PLoS One* 19:e0303694. 10.1371/journal.pone.0303694 38870188 PMC11175485

[B70] Musella K. E. DiFonte M. C. Michel R. Stamates A. Flannery-Schroeder E. (2023). Emotion regulation as a mediator in the relationship between childhood maltreatment and symptoms of social anxiety among college students. *J. Am. Coll. Health* 24 1–8. 10.1080/07448481.2024.2325926 38466343

[B71] Myung I. J. (2003). Tutorial on maximum likelihood estimation. *J. Math. Psychol.* 47 90–100. 10.1016/S0022-2496(02)00028-7

[B72] Nazari M. Shabani R. Dalili S. (2020). The effect of concurrent resistance-aerobic training on serum cortisol level, anxiety, and quality of life in pediatric type 1 diabetes. *J. Pediatric Endocrinol. Metab.* 33 599–604. 10.1515/jpem-2019-0526 32284450

[B73] O’Donnell A. W. Redmond G. Gardner A. A. (2024). Extracurricular activity participation, school belonging, and depressed mood: A test of the compensation hypothesis during adolescence. *Appl. Dev. Sci.* 28 596–611. 10.1080/10888691.2023.2260745

[B74] Panza M. J. Graupensperger S. Agans J. P. Dor I. Vella S. A. Evans M. B. (2020). Adolescent sport participation and symptoms of anxiety and depression: A systematic review and meta-analysis. *J. Sports Exerc. Psychol.* 42 201–218. 10.1123/jsep.2019-0235 32438339 PMC7679280

[B75] Pelissolo A. Abou Kassm S. Delhay L. (2019). Therapeutic strategies for social anxiety disorder: Where are we now? *Expert Rev. Neurotherapeutics* 19 1179–1189. 10.1080/14737175.2019.1666713 31502896

[B76] Piccirillo M. L. Rodebaugh T. L. (2022). Personalized networks of social anxiety disorder and depression and implications for treatment. *J. Affect. Disord.* 298 262–276. 10.1016/j.jad.2021.10.034 34699851 PMC8690310

[B77] Qi Y. Yin Y. Wang X. Zou Y. Liu B. (2024). Autonomous motivation, social support, and physical activity in school children: Moderating effects of school-based rope skipping sports participation. *Front. Public Health* 12:1295924. 10.3389/fpubh.2024.1295924 38327571 PMC10847259

[B78] Ranta K. Junttila N. Laakkonen E. Uhmavaara A. La Greca A. Niemi P. (2012). Social anxiety scale for adolescents (SAS-A): Measuring social anxiety among finnish adolescents. *Child Psychiatry Hum. Dev.* 43 574–591. 10.1007/s10578-012-0285-2 22350460

[B79] Rapee R. M. Magson N. R. Forbes M. K. Richardson C. Johnco C. Oar E. et al. (2022). Risk for social anxiety in early adolescence: Longitudinal impact of pubertal development, appearance comparisons, and peer connections. *Behav. Res. Therapy* 154:104126. 10.1016/j.brat.2022.104126 35642989

[B80] Ren Y. Li M. (2020). Influence of physical exercise on social anxiety of left-behind children in rural areas in China: The mediator and moderator role of perceived social support. *J. Affect. Disord.* 266 223–229. 10.1016/j.jad.2020.01.152 32056881

[B81] Rinta-Antila K. Koski P. Heinonen O. J. (2023). Educational and family-related determinants of organized sports participation patterns from adolescence to emerging adulthood: A four-year follow-up study. *Int. J. Health Promot. Educ.* 61 317–331. 10.1080/14635240.2022.2116943

[B82] Sartika Y. Harahap D. A. Rahmi J. Hindratni F. Sari S. I. P. (2023). Primigravid patients’ cortisol level and anxiety level toward childbirth with pregnancy exercises and healing touch. *J. Pharm. Negative Results* 14, 3207–3216.

[B83] Sasayama K. Nonoue K. Tada T. Adachi M. (2024). Continuation of extracurricular sports activities contributes to higher physical fitness and maintaining academic performance. *Sport Sci. Health* 20 1243–1252. 10.1007/s11332-024-01200-0

[B110] Shang Y. Chen S.-P. Liu L.-P. (2023). The role of peer relationships and flow experience in the relationship between physical exercise and social anxiety in middle school students. *BMC Psychol.* 11:428. 10.1186/s40359-023-01473-z 38057828 PMC10702045

[B84] Sharifi K. D. Sidiqi K. M. Ajmiri M. Y. (2024). The impact of a harmonious sports environment on learning interest. *Sprin J. Arts Humanities Soc. Sci.* 3 20–23. 10.55559/sjahss.v3i4.271

[B85] Sheng X. Wen X. Liu J. Zhou X. Li K. (2024). Effects of physical activity on anxiety levels in college students: Mediating role of emotion regulation. *PeerJ* 12:e17961. 10.7717/peerj.17961 39308821 PMC11416097

[B86] Singh B. Olds T. Curtis R. Dumuid D. Virgara R. Watson A. et al. (2023). Effectiveness of physical activity interventions for improving depression, anxiety and distress: An overview of systematic reviews. *Br. J. Sports Med.* 57 1203–1209. 10.1136/bjsports-2022-106195 36796860 PMC10579187

[B87] Solmaz S. İnan M. Şahin M. Y. (2025). The moderating effects of physical activity on social anxiety and sleep disturbance: Managing gaming disorder in young e-sports players. *Front. Public Health* 13:1544044. 10.3389/fpubh.2025.1544044 39980917 PMC11841710

[B88] Spence S. H. Lawrence D. Zubrick S. R. (2022). Anxiety trajectories in adolescents and the impact of social support and peer victimization. *Res. Child Adolesc. Psychopathol.* 50 795–807. 10.1007/s10802-021-00887-w 35031918

[B89] Sterba S. K. (2014). Fitting nonlinear latent growth curve models with individually varying time points. *Struct. Equ. Model. Multidisciplinary J.* 21 630–647. 10.1080/10705511.2014.919828

[B90] Stone G. A. (2018). The neuroscience of self-efficacy: Vertically integrated leisure theory and its implications for theory-based programming. *J. Outdoor Recreation Educ. Leadersh.* 10 87–96. 10.18666/JOREL-2018-V10-I2-7606

[B91] Tehrani H. D. Yamini S. Vazsonyi A. T. (2024). Parenting styles and big five personality traits among adolescents: A meta-analysis. *Pers. Individ. Dif.* 216:112421. 10.1016/j.paid.2023.112421

[B92] Urbán D. J. La Greca A. M. García-Fernández J. M. Ingles C. A. (2024). bibliometric analysis on adolescent social anxiety and psychoeducational variables in Web of Science 2002–2021. *J. General Psychol.* 151 1–20. 10.1080/00221309.2022.2161982 37233616

[B93] Vasheghani-Farahani A. Tahmasbi M. Asheri H. Ashraf H. Nedjat S. Kordi R. et al. (2011). The persian, last 7-day, long form of the international physical activity questionnaire: Translation and validation study. *Asian J. Sports Med.* 2 106–116. 10.5812/asjsm.34781 22375226 PMC3289200

[B94] Villalongo Andino M. Garcia K. M. Richey J. A. (2024). Can dialectical behavior therapy skills group treat social anxiety disorder? A brief integrative review. *Front. Psychol.* 14:1331200. 10.3389/fpsyg.2023.1331200 38259541 PMC10800915

[B95] Wang F. Ma X. Zhao L. Li T. Fu Y. Zhu W. et al. (2024). The influence of genetic and environmental factors on anxiety among chinese adolescents: A twin study. *J. Genet. Psychol.* 185 415–426. 10.1080/00221325.2024.2319235 38456243

[B96] Wang J. Wu S. Chen X. Xu B. (2024). Impact of awareness of sports policies, school, family, and community environmental on physical activity and fitness among children and adolescents: A structural equation modeling study. *BMC Public Health* 24:2298. 10.1186/s12889-024-19795-x 39256716 PMC11389504

[B97] Wang M. T. Eccles J. S. (2012). Social support matters: Longitudinal effects of social support on three dimensions of school engagement from middle to high school. *Child Dev.* 83 877–895. 10.1111/j.1467-8624.2012.01745.x 22506836

[B98] Wang Q. Dowsey M. M. Woodward-Kron R. Brien P. Hawke L. Bunzli S. (2021). Contributing factors to decline in physical activity among adolescents: A scoping review. *Malaysian J. Soc. Sci. Humanities* 6 447–463. 10.47405/mjssh.v6i9.998

[B99] Wang Y. (2024). The role of competitive vs. recreational group sports in reducing social anxiety among Chinese teenagers. *Stud. Sports Sci. Phys. Educ.* 2, 34–42.

[B100] Wu X. Qi J. Zhen R. (2021). Bullying victimization and adolescents’ social anxiety: Roles of shame and self-esteem. *Child Ind. Res.* 14 769–781. 10.1007/s12187-020-09777-x

[B101] Yang P. Pachman S. L. Schlomer G. L. Edin J. (2024). Direct and indirect longitudinal associations of mother and father engagement in middle childhood on adolescent externalizing and internalizing behaviors. *J. Youth Adolesc.* 53 1832–1846. 10.1007/s10964-024-01982-z 38600264 PMC12094873

[B102] Yang Y. (2024). Promoting youth participation in sports: An analysis of home-school-community approach. *Int. J. Educ. Humanities* 14 260–265. 10.54097/341dwa27

[B103] Yin X. Peng J. Zhu K. Li Z. (2024). Developmental trajectories of hyperactive behavior in children from low-income families: A latent variable growth model analysis. *Curr. Psychol.* 43, 13608–13618.

[B105] Zhang X. Li W. Wang J. (2022). Effects of exercise intervention on students’ test anxiety: A systematic review with a meta-analysis. *Int. J. Environ. Res. Public Health* 19:6709. 10.3390/ijerph19116709 35682293 PMC9180005

[B106] Zhang Y. Wen Z. Zhu Y. Guan G. (2024). Effects of physical exercise on body esteem among females: A meta-analysis. *BMC Public Health* 24:3387. 10.1186/s12889-024-20861-7 39639264 PMC11622481

[B107] Zhang Z. Yang R. Sun G. Wang Y. (2024). Impact of crossfit training programs on the physical health and sociogenic somatic anxiety of adolescents. *Iran J. Public Health* 53 1588–1597. 10.18502/ijph.v53i7.16053 39086416 PMC11287592

[B108] Zheng Y. Ye Y. (2022). Prediction of cognitive-behavioral therapy using deep learning for the treatment of adolescent social anxiety and mental health conditions. *Sci. Programm.* 2022:3187403. 10.1155/2022/3187403

[B109] Zhou J. Wang L. Zhu D. Gong X. (2024). Social anxiety and peer victimization and aggression: Examining reciprocal trait-state effects among early adolescents. *J. Youth Adolesc.* 53 701–717. 10.1007/s10964-023-01920-5 38097883

